# A coupled magneto–mechanical–thermal constitutive model for transversely isotropic magnetorheological elastomers verified by experimental validation

**DOI:** 10.1177/1045389X261432516

**Published:** 2026-03-24

**Authors:** Amin Saber, Ramin Sedaghati

**Affiliations:** 1 Department of Mechanical, Industrial and Aerospace, Concordia University, Montreal, QC, Canada

**Keywords:** magnetorheological elastomer, numerical simulation, solid mechanics, experiment

## Abstract

Magnetorheological elastomers, as a class of smart functional materials, have attracted considerable attention in various engineering and biomedical applications. Transversely isotropic MREs containing chain-like magnetic particle structures particularly exhibit enhanced mechanical and actuation properties compared with their isotropic counterparts. Employing nonlinear continuum mechanics framework together with finite strain theory, this study develops a physics-based constitutive model to predict the coupled magneto–mechanical–thermal behavior of such materials. In this formulation, the deformation gradient, magnetic induction, temperature, and particle-chain orientation are treated as independent variables in defining the total Helmholtz free energy function. In addition to the proposed theoretical framework, a series of experimental tests are performed for model validation and parameter identification. The overall behavior of transversely isotropic MREs is subsequently examined under different particle-chain orientations and loading conditions. For the cylindrical MRE specimen, the results show that the material stiffness increases with both magnetic field strength and temperature, and exhibits a peak response at a particle-chain tilt angle of approximately 45° relative to the axial direction.

## Introduction

Magnetorheological elastomers (MREs) are functional composite materials in which magnetic particles are dispersed within an elastomeric matrix, imparting magnetoactive behavior to the material. Depending on the arrangement of the magnetic particles within the matrix, MREs are generally classified as either isotropic, characterized by randomly and uniformly distributed particles, or anisotropic, in which the particles exhibit a preferred directional alignment. When subjected to an external magnetic field, MREs behave as smart functional materials whose mechanical and magnetic properties can be actively tuned by varying the applied loading conditions. This tunable feature makes them promising candidates for a wide range of engineering applications. For instance, the incorporation of MREs in vibration isolators and dampers enables controllable stiffness and damping characteristics, allowing these devices to adapt in real time to varying operating conditions over a wide range of excitation frequencies and amplitudes (Bastola and Li, 2018; Choi and Wereley, 2022; Erenchun et al., 2024; Lin et al., 2023; Sun et al., 2020; Tao et al., 2018). In addition, MREs play a crucial role in the development of soft actuators and soft robotic systems by providing magnetically tunable stiffness and force output within compliant structures. This field-responsive behavior allows soft actuators to dynamically adjust their mechanical response, thereby achieving an effective balance between flexibility and load-bearing capability according to task requirements (Bellelli et al., 2025; Böse et al., 2021; Jadhav et al., 2022; Park et al., 2023; Xu et al., 2024). More recently, attention has extended toward their potential use in biomedical engineering, such as tissue engineering scaffolds (Gonzalez-Rico et al., 2021), targeted drug delivery systems (Cesmeci et al., 2023; Vakil et al., 2022), and cardiovascular surgical devices (Hooshiar et al., 2021). Understanding, characterizing, and accurately modeling the behavior of MREs under various conditions is therefore of critical importance for effective use of these emerging mart materials for design and development of MRE-based adaptive systems and structures.

Extensive experimental investigations have also been conducted to characterize the behavior of MREs with respect to various parameters such as particle size and volume fraction (Li and Sun, 2013; Winger et al., 2019), prestrain (Lejon and Kari, 2009; Vatandoost et al., 2021), type of magnetic particles used (Wu et al., 2010), and effect of particle orientation angle on magnetostrictive performance (Guan et al., 2008). It is also reported that, compared with their isotropic counterparts, anisotropic MREs exhibit a more pronounced magnetorheological (MR) effect (Bellanc and Bossis, 2002; Fakhree et al., 2022). This enhancement arises from the reduced interparticle distance, which strengthens the magnetic interactions between adjacent particles. Studying the effect of MRE matrix, Alam et al. (2021) investigated isotropic and anisotropic NR- and NBR-based MREs and demonstrated that NBR composites exhibit stronger magnetic stiffening, better particle-chain structuring, and enhanced anti-stress-relaxation behavior. However, their study remained empirical and did not provide a predictive framework for field- and orientation-dependent response.

In addition to experimental investigations, most modeling approaches for MREs have employed phenomenological techniques, whose predictive capability is typically restricted to the range of loading conditions used in the experiment for parameter identification. Gong et al. (2022) developed a torque-balance model where particle–particle and chain–chain interactions govern field-induced bending, yielding acceptable agreement with deformation experiments but applying only to slender, suspended chain-like specimens under small rotations. Tian and Nakano (2018) quantified the viscoelastic response of directional MREs with a 45° particle-chain alignment, but did not provide a constitutive model capable of predicting the material behavior under general loading conditions. For a detailed overview of these modeling strategies, readers are referred to the review paper presented in Saber and Sedaghati (2023). Among continuum-based models, which have received comparatively less attention, one of the earliest attempts to describe the behavior of transversely isotropic MREs was made by Bustamante (2010), who developed a physics-based framework using the concept of the Helmholtz free energy function to derive constitutive relations for materials with a preferred chain orientation. However, due to the limited availability of experimental data at that time, the material parameters could not be thoroughly identified. Saxena et al. (2014) extended the mathematical framework to incorporate the magneto-viscoelastic behavior of transversely isotropic MREs by introducing an internal variable into the constitutive equations to account for material time-dependent response. Later, Itskov and Khiêm (2016) proposed a polyconvex free-energy formulation using generalized magneto-elastic invariants and structural tensors, but this framework was purely isothermal with lack of experimental validation in thermally varying and highly anisotropic MREs. Recently, Beheshti et al. (2021) presented a refined continuum model based on a modified Lopez-Pamies energy function to describe the magneto-mechanical response of transversely isotropic MREs, supported by a series of experimental tests for parameter identification.

Experimental observations have demonstrated that temperature exerts a significant influence on both the elastic and viscoelastic responses of MREs (Saber and Sedaghati, 2025c). To capture this effect, a theoretical model incorporating temperature as an independent variable was introduced into the total energy function using the concept of constant heat capacity (Mehnert et al., 2017; Vertechy et al., 2013). More recently, Saber and Sedaghati (2025a) extended this framework and performed experimental investigations to validate their proposed magneto-hyperelastic–thermal coupled model for isotropic MREs. In this study, the temperature range examined was −10°C to 40°C, and the results showed that for an Ecoflex-0010 matrix, an increase in temperature led to higher shear stiffness. However, it has also been reported that the influence of temperature and the magnetorheological effect on the mechanical response of the matrix phase depends strongly on the specific elastomer employed (Liao et al., 2020; Poojary et al., 2024). More specifically, temperature-dependent trends in MRE stiffness are known to be matrix-sensitive and may exhibit either monotonic or non-monotonic behavior, depending on factors such as elastomer chemistry, filler–matrix bonding characteristics, and the applied loading conditions (Agirre-Olabide et al., 2015; Li et al., 2016; Wan et al., 2019; Zhang et al., 2011).

Although several studies have proposed physics-based models to describe the behavior of MREs, most have focused exclusively on their isothermal magneto-mechanical coupling, with relatively few addressing the combined magneto-mechanical–thermal interactions. Moreover, the limited number of existing thermo-magneto-mechanical models have primarily been developed for isotropic MREs. Motivated by this research gap, the present study aims to develop a coupled magneto-mechanical–thermal physics-based model capable of predicting the response of transversely isotropic MREs under various loading conditions, supported by experimental validation.

Consequently, the model is applicable to a broad class of MREs that can be idealized as (i) nearly incompressible, (ii) characterized by a single preferred direction associated with particle-chain alignment (transverse isotropy), and (iii) assuming quasi-static condition, magnetic particles with negligible remnant magnetization, an effectively linear magnetic response, and insignificant magnetostriction. Provided that these assumptions are satisfied, the proposed framework is capable of describing the MRE response under various combinations of magnetic, thermal, and mechanical loading conditions.

The rest of this paper is organized as follows. Section 2 outlines the fabrication procedure and experimental testing methods. Section 3 presents the fundamental concepts and governing equations used to describe the coupled magneto-mechanical–thermal deformation response of transversely isotropic MREs. The analytical results and model verification are discussed in Section 4, followed by the concluding remarks in Section 5.

## Experiment

To characterize the response of transversely isotropic MREs and to validate the proposed model, a series of experimental tests were performed. The fabrication process and testing procedures are presented in this section. The primary objective of these experiments is to investigate the coupled magneto–mechanical–thermal behavior of a fabricated transversely isotropic MRE cylindrical specimen subjected to torque–twist loading.

### MRE sample fabrication

**Figure 1.**
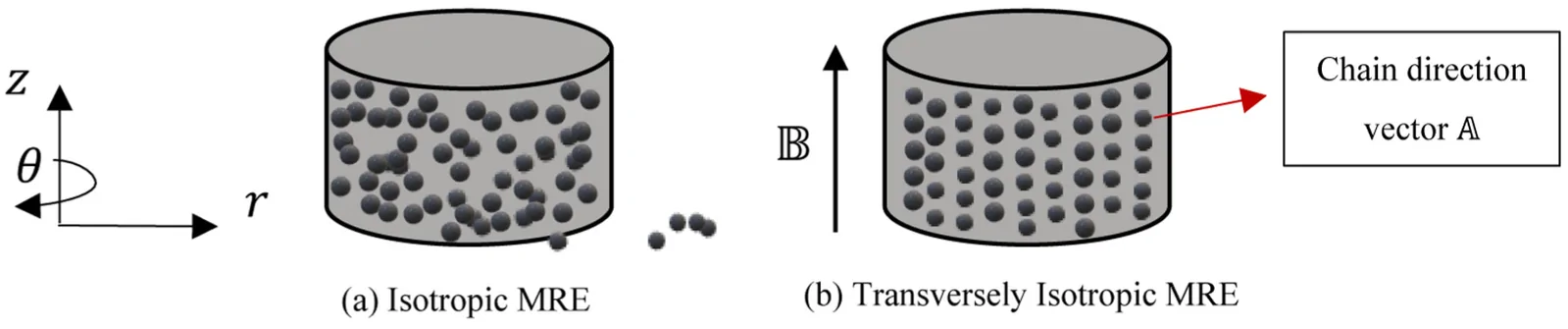
The schematic illustration of: (a) isotropic MRE cylinder and (b) transversely isotropic MRE cylinder with chain orientation of vector A .

**Figure 2.**
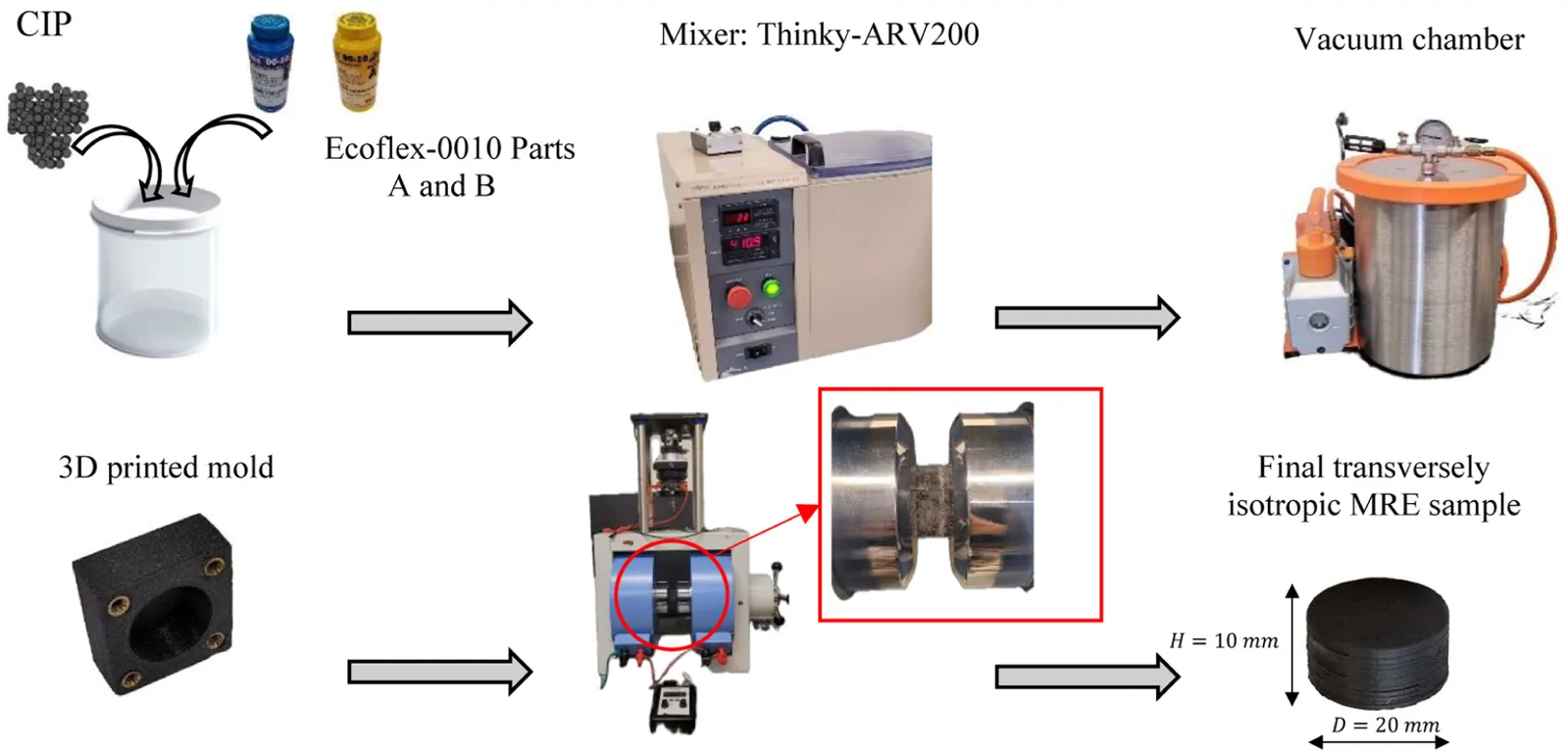
Schematic summary of the fabrication process for the transversely isotropic MRE sample.

MREs generally consist of two main components: an elastomeric matrix and dispersed magnetic particles. In some cases, additives are incorporated to facilitate fabrication or to improve the mechanical and magnetic properties of the final composite. In this study, to fabricate a transversely isotropic MRE cylindrical sample (Figure 1), a commercial silicone elastomer, Ecoflex-0010™ with a Shore hardness of 10, was used as the host matrix, while micron-sized BASF soft carbonyl iron powder (CIP) served as the ferromagnetic phase. To fabricate the transversely isotropic MRE samples, parts A and B of Ecoflex-0010 were mixed at a 1:1 ratio, and CIP was added at a volume fraction of 25%. The mixture was then blended using a planetary mixer operating at 2000 rpm for 45 s to ensure uniform dispersion of the particles. To remove trapped air bubbles, the mixture was subsequently placed in a vacuum chamber at a pressure of 27 in Hg for approximately 3 min and then slowly poured into a 3D-printed mold designed to produce cylindrical samples with a height of 10 mm and a diameter of 20 mm. To induce a chain-like alignment of the magnetic particles, the mold containing the uncured mixture was positioned between two magnetic poles and exposed to a magnetic field of 0.95 T for 2 h. This process ensured the formation of particle chains aligned along the direction of the applied field, corresponding to the cylinder’s axial (*z*) direction. Consequently, the particle-chain direction is represented by a single, uniform structural unit vector A defined in the reference configuration and assumed constant throughout the specimen, implying that no spatially varying chain orientation is considered within the MRE sample. It should be noted that, in practice, individual particle chains are not perfectly parallel; nevertheless, a single dominant mean orientation exists, which is able to precisely capture the behavior of transversely isotropic MREs. Figure 2 provides an overview of the fabrication process for the transversely isotropic MREs.

### Testing protocols

**Figure 3.**
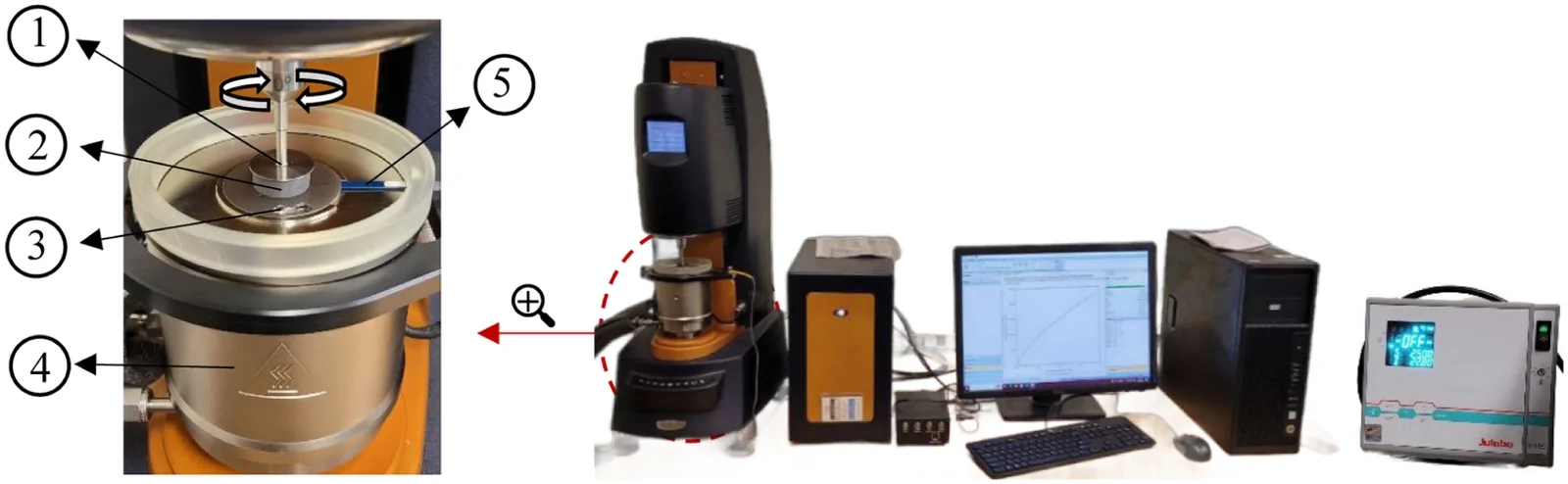
Test setup configuration: 1—upper plate geometry, 2—MRE sample, 3—lower plate geometry, 4—magneto-rheology accessory, 5—hall probe.

To validate the proposed constitutive model and identify the material parameters, the torque–twist response of transversely isotropic MREs under coupled magneto–mechanical–thermal loading was examined using an advanced rotational rheometer (Discovery HR-3, TA Instruments). The device is equipped with a magnetorheology accessory and integrated with a temperature control system (Julabo F32-HE high-capacity refrigerated/heating circulator), enabling simultaneous control of magnetic induction from 0 to 1 T and temperature from −10°C to 70°C. During testing, the MRE specimen was positioned between the upper and lower plates, with the lower plate remaining fixed while the upper plate applied the mechanical deformation. The temperature control system maintained the desired thermal condition at the lower plate. Because the MRE sample height was 10 mm, it was verified that the magnetic induction varied by approximately 25% between the bottom and top surfaces of the cylindrical specimen. To account for this nonuniformity, the average magnetic induction across the sample height was used in the modeling framework, and it was verified that the resulting approximation introduced only a small error relative to the experimental measurements. After the specimen was positioned, an axial compressive force was applied by slightly reducing the gap between the plates to prevent slippage during twisting. The influence of this axial preload was incorporated into the constitutive model through an axial stretch variable in the governing equations. The final gap value was measured after considering for any potential thermal expansion. Figure 3 illustrates the main components of the experimental setup.

## Fundamental governing equations

The developed model is applicable to a broad class of MREs that can be idealized as (i) nearly incompressible, (ii) characterized by a single preferred direction associated with particle-chain alignment, corresponding to transverse isotropy, and (iii) operating under quasi-static conditions with magnetic particles exhibiting negligible remnant magnetization, an effectively linear magnetic response, and insignificant magnetostriction. Provided that these assumptions are satisfied, the proposed framework is capable of describing the MRE response under various combinations of magnetic, thermal, and mechanical loading conditions. Accordingly, finite strain theory is employed to model the constitutive behavior of nonlinear continua undergoing large deformations. To this end, the kinematics of deformation must first be established. Assuming that MREs behave as continuous media, their deformation can be represented in two distinct configurations: the reference configuration and the current configuration, which are related through the deformation gradient tensor F . Accordingly, the strain measures can be defined in terms of either the right or the left Cauchy–Green deformation tensors, expressed as follows:

(1) $$c=F^{T}F$$

(2) $$b={\mathrm{FF}}^{T}$$

here, c and b are symmetric, positive-definite tensors known as the right and left Cauchy–Green deformation tensors, respectively. To derive the general constitutive relations, the formulation is based on the first and second laws of thermodynamics. Accordingly, the governing equation can be expressed as follows:

(3) $$\begin{array}{c} -\rho \left(\frac{\partial \psi }{\partial T}+s\right)\overset{\cdot }{T}+\left({\left(F^{-1}\sigma \right)}^{T}-\rho \frac{\partial \psi }{\partial F}\right):\\ \overset{\cdot }{F}-\left(\rho \frac{\partial \psi }{\partial B}+M_{e}\right).\overset{\cdot }{B}-\left(\frac{1}{T}\;\mathrm{grad}\;T\right)\;.\;Q\geq 0 \end{array}$$

In this expression, ψ , B , s , T , Q , and $M_{e}$ represent the Helmholtz free energy function, magnetic induction vector, specific entropy, absolute temperature, heat flux vector, and effective magnetization vector, respectively. Following Clausius–Duhem argument, the following constitutive relations are subsequently obtained:

(4) $$s=-\frac{\partial \psi }{\partial T}$$

(5) $$\sigma =\rho F{\left(\frac{\partial \psi }{\partial F}\right)}^{T}$$

(6) $$M_{e}=-\rho \frac{\partial \psi }{\partial B}$$

(7) $$-\left(\frac{1}{T}\;\mathrm{grad}\;T\right)\;.\;Q\geq 0$$

To incorporate the effect of temperature into the total Helmholtz free energy function, the assumption of a constant specific heat capacity is employed. Accordingly, the specific heat at constant volume, denoted by $c_{0}$ , is defined as $c_{0}=T\left(-\frac{\partial^{2}\psi }{\partial T^{2}}\right)=\mathrm{constant}$ . By integrating this relation twice with respect to temperature, the modified form of the total Helmholtz free energy function is obtained as follows:

(8) $$\begin{array}{c} \psi \left(F,\;B,A,\;T\right)=c_{0}\left[T-T_{0}-T\ln\left(\frac{T}{T_{0}}\right)\right]-\left(T-T_{0}\right)\\ \;W_{1}\left(F,\;B\right)+W_{2}\left(F,\;B,A\right) \end{array}$$

With the chain-orientation vector A introduced in equation (8), the total energy function derived above consists of three main contributions: the purely thermal term, the thermo-mechanical coupling term, and the isothermal mechanical term. Following the arguments presented by Mehnert et al. (2017), the function $W_{1}$ can be additively decomposed into mechanical and magneto-mechanical components. The mechanical part is expressed as $M_{1}\left(F\right)=3\;\epsilon \;\beta \;\ln\;(J)$ and the magneto-mechanical coupling term is given by $M_{2}\left(F,\;B,A\right)=-\frac{1}{T_{0}}W_{2}\left(F,\;B,A\right)$ ; where ϵ and β denote the specific bulk modulus and the linear thermal expansion coefficient, respectively. This approach reflects the natural separation between the mechanical and magneto-mechanical mechanisms represented in the Helmholtz free energy, and it preserves the standard forms of the entropy and stress relations when substituted into the Clausius–Duhem inequality. The additive decomposition also ensures full compatibility with the second law of thermodynamics. Thus, the total Helmholtz free energy function in equation (8), can be written in the following simplified form:

(9) $$\begin{array}{c} \psi \left(F,\;B,A,\;T\right)=c_{0}\left[T-T_{0}-T\ln\left(\frac{T}{T_{0}}\right)\right]\\ \;-\left(T-T_{0}\right)M_{1}\left(F\right)+\frac{T}{T_{0}}W_{2}\left(F,\;B,A\right) \end{array}$$

**Figure 4.**
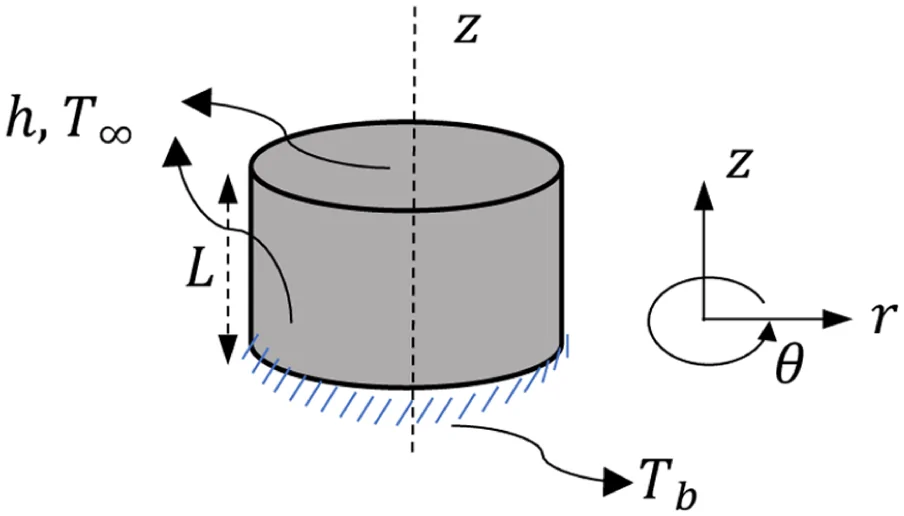
The thermal boundary conditions.

It is noted that in the formulation, the temperature T may itself be a spatially varying function of the material coordinates, representing the temperature distribution within the MRE. The term $W_{2}$ corresponds to the isothermal magneto-mechanical coupling energy function. The objective here is therefore to determine the temperature field T(r,θ,z) and the function $W_{2}$ , respectively. Considering the thermal boundary conditions used in the experiments—namely, a fixed temperature at the bottom face of the cylindrical specimen and free convection on all other surfaces (see Figure 4)—the governing heat equation can be solved accordingly. It should be noted that the top surface is in contact with a thin stainless-steel plate, however, the thermal conductivity of steel is significantly higher than that of the MRE, such that the steel plate behaves effectively as an isothermal body. Therefore, it is a justified simplification to treat the top surface of the elastomer as being subject to free convection to ambient air, identical to the lateral surface, while only the bottom surface is maintained at a prescribed temperature. Under these assumptions, the solution to the heat equation can be expressed as follows:

(10) $$\bar{T}\left(r,z\right)=\sum_{n=1}^{\infty }E_{n}J_{0}\left(\lambda_{n}r\right)\sinh\;\left(\lambda_{n}z\right)-\frac{h}{\mathrm{hL}+\kappa_{\mathrm{eff}}}z+1$$

with

(11) $$\kappa_{\mathrm{eff}}=\kappa_{m}\frac{2\kappa_{m}+\kappa_{f}+2\phi \left(\kappa_{f}-\kappa_{m}\right)}{2\kappa_{m}+\kappa_{f}-\phi \left(\kappa_{f}-\kappa_{m}\right)}$$

where $\bar{T}=\frac{T-T_{\infty }}{T_{b}-T_{\infty }}$ and we have:

(12) $$E_{n}=\frac{\frac{2}{L}\left[\frac{h}{hL+\kappa_{eff}}\left(\frac{L}{\lambda_{n}}\cosh\left(\lambda_{n}L\right)-\frac{1}{\lambda_{n}^{2}}\sinh\left(\lambda_{n}L\right)\right)-\frac{1}{\lambda_{n}}\left(\cosh\left(\lambda_{n}L\right)-1\right)\right]}{\frac{\kappa_{eff}}{h}\lambda_{n}J_{1}\left(\lambda_{n}R\right)-J_{0}\left(\lambda_{n}R\right)}$$

where in this expression, h denotes the convection heat transfer coefficient, and $\kappa_{\mathrm{eff}}\;$ represents the effective thermal conductivity, which accounts for the contributions of both the magnetic particles $\left(\kappa_{f}\right)$ and the elastomeric matrix $\left(\kappa_{m}\right)$ thermal conductivities. The corresponding values of the material properties used in this study are provided in Saber and Sedaghati (2025a). $J_{0}$ and $J_{1}$ are the Bessel functions of the first kind of order zero and one, respectively. $\lambda_{n}$ denotes the characteristic values obtained from the solution of the characteristic equation $\frac{\kappa_{\mathrm{eff}}\lambda }{h}=\tanh(\lambda z)$ . The next step is to define the isothermal magneto-mechanical energy function $W_{2}$ . According to Cayley-Hamilton and Rivlin representation theorems, it can be concluded that in general the constitutive relations like stress-strain or free-energy function which is the case here, can be written as functions of invariants of left or right Cauchy-Green deformation tensors (Holzapfel, 2002). It was shown that for an isotropic pure mechanical material, the energy function can be written in terms of three invariants of tensor c , and to account for any preferred directions, we need to add more invariants (Spencer, 1971). In this regard, the total of 10 invariants that are needed to describe the transversely magneto-mechanical behavior of MREs, are listed below:

(13-1) $$\begin{array}{c} I_{1}=\mathrm{trace}(c)\;I_{2}=0.5\times \left(\mathrm{trace}{\left(c\right)}^{2}-\mathrm{trace}\left(c^{2}\right)\right)\\ \;I_{3}=\det(c) \end{array}$$

(13-2) $$I_{4}=B_{0}\;\cdot \;B_{0}\;I_{5}=B\cdot (c\;⊗B_{0})\;I_{6}=B_{0}\cdot (c^{2}\;⊗B_{0})$$

(13-3) $$\begin{array}{c} I_{7}=A_{0}\cdot (c\;⊗A_{0})\;I_{8}=A_{0}\cdot (c^{2}\;⊗A_{0})\;I_{9}=A_{0}\cdot \\ \;B_{0}\;I_{10}=A_{0}\cdot (c\;⊗B_{0}) \end{array}$$

Therefore, based on the theory of invariants and under the assumption of material incompressibility ( J=1 ), the general form of the total energy function presented in equation (9) can be simplified as follows:

(14) $$\begin{array}{c} \psi \left(I_{1},I_{2},I_{4},I_{5},I_{6},I_{7},I_{8},I_{9},I_{10},T\right)=c_{0}\\ \left[T-T_{0}-T\ln\left(\frac{T}{T_{0}}\right)\right]+\frac{T}{T_{0}}W_{2}\left(I_{1},I_{2},I_{4},I_{5},I_{6},I_{7},I_{8},I_{9},I_{10}\right) \end{array}$$

By proposing an appropriate form of the energy function $W_{2}$ and subsequently applying the constitutive relations, the objectivity and symmetry requirements of the formulation are ensured. Next, the stress–strain response of the material can be derived to describe the overall behavior of the MRE. This formulation will be further discussed in the following section, where the solution of a representative boundary value problem is presented.

## Results and discussion

The objective of this section is to determine the torque–twist response of the fabricated transversely isotropic MRE specimen. Before this can be accomplished, the function $W_{2}$ , must be defined. In its most general form, $W_{2}$ should be formulated to incorporate both the purely mechanical contribution and the magneto–mechanical coupling effects. Accordingly, $W_{2}$ can be expressed as the sum of three components, $W_{2,\mathrm{mech}}$ , $W_{2,\mathrm{mag}-\mathrm{mech}}$ , and $W_{2,\mathrm{mag}}$ . In this framework, a modified Mooney–Rivlin hyperelastic energy function is adopted for $W_{2,\mathrm{mech}}$ , as its accuracy and robustness have been demonstrated in previous studies (Saber and Sedaghati, 2025b). Thus, $W_{2,\mathrm{mech}}$ can be written as follows:

(15) $$\begin{array}{c} W_{2,\mathrm{mech}}=\mu_{\mathrm{eff}}\left(I_{1}-3\right)+C_{12}{\left(I_{1}-3\right)}^{2}+C_{22}{\left(I_{2}-3\right)}^{2}\\ \;+\gamma_{1}{\left(I_{7}-1\right)}^{2}+0.5\times \gamma_{2}{\left(2I_{8}-I_{7}-1\right)}^{2} \end{array}$$

In this formulation, $C_{12}$ , $C_{22}$ , $\gamma_{1}$ , and $\gamma_{2}$ are material constants. The quantity $\mu_{\mathrm{eff}}\;$ represents the initial effective structural shear stiffness of the material, which may vary depending on the applied loading conditions. A practical approach to incorporate these effects into the model is to express $\mu_{\mathrm{eff}}$ as a function of temperature, magnetic induction, and axial stretch. Using a multiplicative interaction form, $\mu_{\mathrm{eff}}$ can be written as follows:

(16) $$\begin{array}{c} \mu_{\mathrm{eff}}\left(B,T,\lambda_{z}\right)=\left[G_{\mathrm{ref}}\left(1+\alpha_{1}\tanh\;\left(\frac{B^{2}}{\alpha_{2}}\right)\right)\right]\\ \;\left[1+\beta_{1}(T-T_{\mathrm{ref}})+\beta_{2}(T-T_{\mathrm{Ref}}{)}^{2}\right]\\ \;(1+\eta_{1}\left(1-\lambda_{z}\right)+\eta_{2}{\left(1-\lambda_{z}\right)}^{2} \end{array})$$

In this expression, $G_{\mathrm{ref}}$ denotes the reference shear modulus measured at the reference temperature $T_{\mathrm{ref}}=294{}^{\circ}K$ . The parameters $\alpha_{1},\alpha_{2},\beta_{1},\beta_{2},\eta_{1}\;\mathrm{and}\;\eta_{2}$ are material constants which are determined in an overdetermined manner using torque–twist data, such that the number of experimental data points are more than the number of fitted parameters, ensuring robustness of the identification process. In this regard, as listed in Table 1, a total of 26 experiments were conducted to systematically examine the effects of temperature, magnetic induction, and axial stretch on the effective shear modulus $G_{\mathrm{eff}}$ . The values of identified material constants are then listed in Table 2.

**Table 1.** Test conditions used in parameter identification.

Temperature °C/(°K)	Magnetic induction (T)	Axial stretch (-)	Effective shear modulus (kPa)
−10 (263)	0	0.97	139.2
−5 (268)	0	0.9	151.8
0 (273)	0	0.9	159.2
10 (283)	0	0.9	179.7
21 (294)	0	0.9	188.6
30 (303)	0	0.9	193.2
40 (313)	0	0.9	195.7
50 (323)	0	0.9	199.1
21 (294)	0	0.96	139.6
21 (294)	0.1	0.96	190.7
21 (294)	0.2	0.96	307.9
21 (294)	0.3	0.96	476
21 (294)	0.4	0.96	695.7
21 (294)	0.5	0.96	907.1
21 (294)	0.6	0.96	1027
21 (294)	0.7	0.96	1055
21 (294)	0	1	116.8
21 (294)	0	0.99	118
21 (294)	0	0.98	123
21 (294)	0	0.97	130
21 (294)	0	0.96	147
21 (294)	0	0.95	180.8
21 (294)	0	0.94	225.1
21 (294)	0	0.93	271.9
21 (294)	0	0.92	324.1
21 (294)	0	0.91	382.7

**Table 2.** Identified parameters for the effective shear modulus model.

$G_{\mathrm{ref}}$ (kPa)	$\alpha_{1}$ (-)	$\alpha_{2}$ ( $T^{2}$ )	$\beta_{1}$ ( ${}^{\circ}K^{-1}$ )	$\beta_{2}$ ( ${}^{\circ}K^{-2}$ )	$\eta_{1}$ (−)	$\eta_{2}$ (−)
116.89	6.24	$2.48\times 10^{-1}$	$5\times 10^{-3}$	$-10^{-4}$	−6.87	362.08

**Figure 5.**
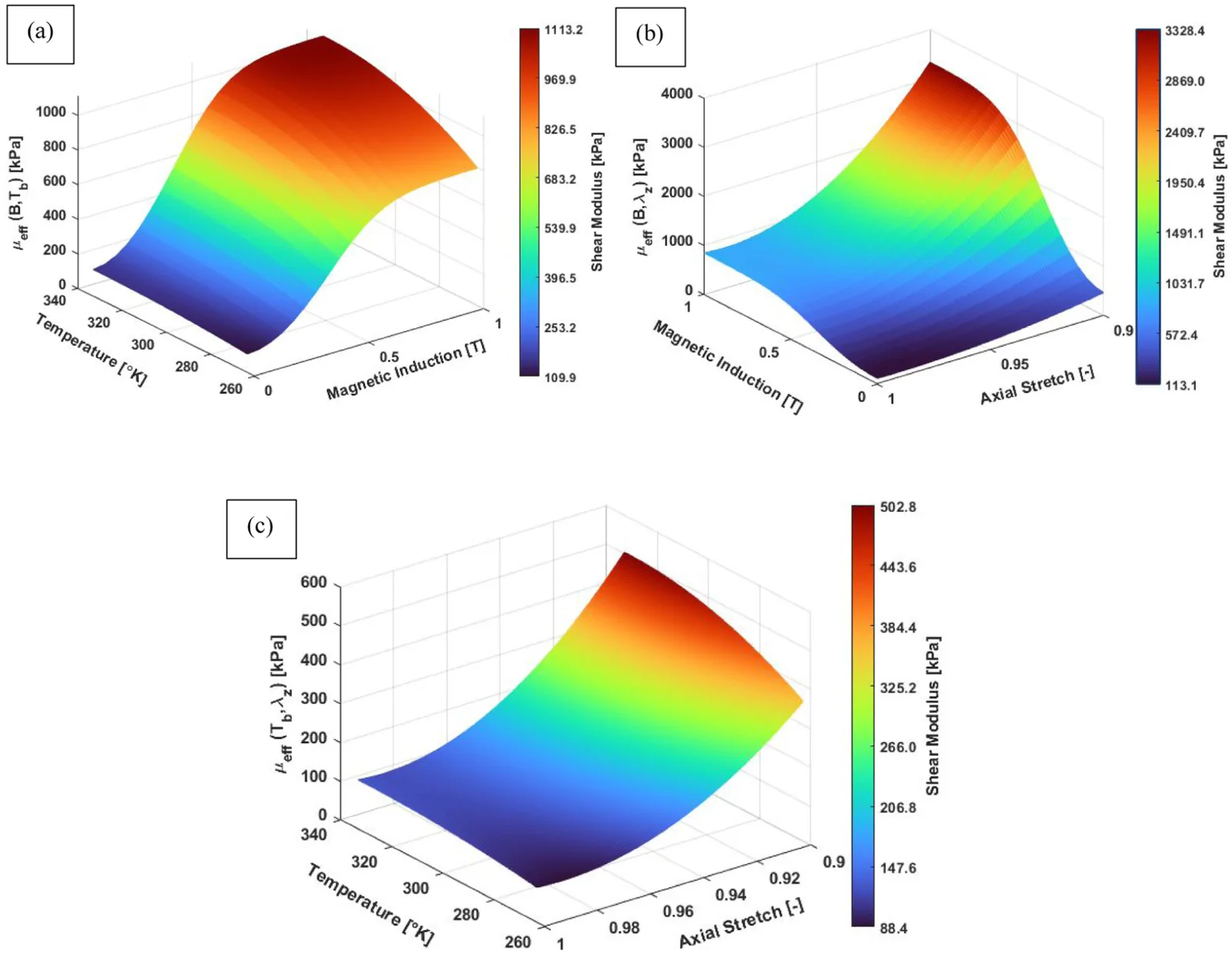
Variation of the effective shear modulus with respect to the independent parameters $B,\;T\;\mathrm{and}\;\lambda_{z}$ : (a) dependence of the effective shear modulus on magnetic induction and temperature, (b) dependence on magnetic induction and axial stretch, and (c) dependence on temperature and axial stretch.

As a result, the variation of the effective shear modulus $\mu_{\mathrm{eff}}\;$ with respect to magnetic induction, temperature, and axial stretch is illustrated in Figure 5.

Based on these results, it can be seen that the external magnetic induction and the axial stretch exert the most pronounced influence on the effective shear modulus. This observation also allows the temperature effect to be treated in a simplified manner when no significant temperature gradient exists within the MRE specimen—that is, when the temperature can be reasonably taken as the average value between the upper and lower surfaces of the cylindrical sample.

As shown in Figure 5, the effective shear modulus also increases with temperature. This behavior can be explained by considering two competing mechanisms through which temperature influences the stiffness of elastomeric materials: entropy-related and enthalpy-related contributions (Li et al., 2016; Saber and Sedaghati, 2025c). The entropy-driven mechanism is associated with ideal rubber elasticity, for which the shear modulus follows the relation G=nkT , where T is the absolute temperature, k is the Boltzmann constant, and n denotes the number of polymer chains per unit volume. In this case, increasing temperature enhances the thermal agitation of polymer chains, leading to a higher entropic restoring force and, consequently, an increase in shear stiffness. In contrast, from a mechanical perspective, elevated temperatures may weaken matrix–matrix and matrix–filler interactions, which tends to reduce the material stiffness. The observed temperature-dependent response therefore results from a competition between these two mechanisms, with the dominant contribution determining whether the shear modulus increases or decreases with temperature.

Next, for the magneto-mechanical contribution of the energy function, $W_{2,\mathrm{mag}-\mathrm{mech}}$ , the following formulation is proposed:

(17) $$\begin{array}{c} W_{2,\mathrm{mag}-\mathrm{mech}}=0.5\times \xi_{1}{\left(I_{5}-I_{4}\right)}^{2}+0.5\times \xi_{2}\\ \;{\left(I_{6}-2I_{5}+I_{4}\right)}^{2}+0.5\times \xi_{3}{\left(I_{10}-I_{9}\right)}^{2} \end{array}$$

The proposed form of the magneto-mechanical energy function, together with the mechanical term, enables the model to account for the magneto-mechanical contribution to the stress response. Each term is formulated in a proper manner so that the magnetostriction vanishes when no mechanical loading is applied ( F=I ), which is consistent with the behavior of MREs with soft iron particles. Consequently, for this particular MRE and under the assumption of negligible magnetostriction, the coefficients $\xi_{1},\xi_{2}$ , and $\xi_{3}$ are taken to be zero. This ensures that the energy function remains magnetostriction-free at zero strain even when a non-zero magnetic induction field is applied. Any field-induced deformation arises solely through Maxwell stress at the boundaries. Next, assuming linear magnetic behavior with no hysteresis, the relationship between B and M can be given by $B=\mu_{0}\mu^{r}H$ where $\mu^{r}$ is second order relative permeability tensor. For a transversely isotropic medium with the symmetry axis along z (unit vector A) , one can make use of equation (6), and define the magnetic energy function as below (Krupka, 2018):

(18) $$\psi^{\mathrm{mag}}\left(B,A\right)=-\zeta_{1}B\cdot B-\zeta_{2}{\left(A\cdot B\right)}^{2}$$

Using the experimental data reported, the relative permeabilities in the directions parallel and perpendicular to the particle-chain orientation were measured as $\mu_{∥}^{r}=2.3\;$ and $\mu_{⊥}^{r}=1.35$ , respectively (Beheshti et al., 2021). Accordingly, the constants $\zeta_{1}\;$ and $\zeta_{2}$ can be determined as follows:

(19) $$\zeta_{1}=\frac{1}{2\mu_{0}}\left(1-\frac{1}{\mu_{⊥}^{r}}\right)\;,\;\mathrm{and}\;\zeta_{2}=\frac{1}{2\mu_{0}}\left(\frac{1}{\mu_{⊥}^{r}}-\frac{1}{\mu_{∥}^{r}}\right)$$

**Figure 6.**
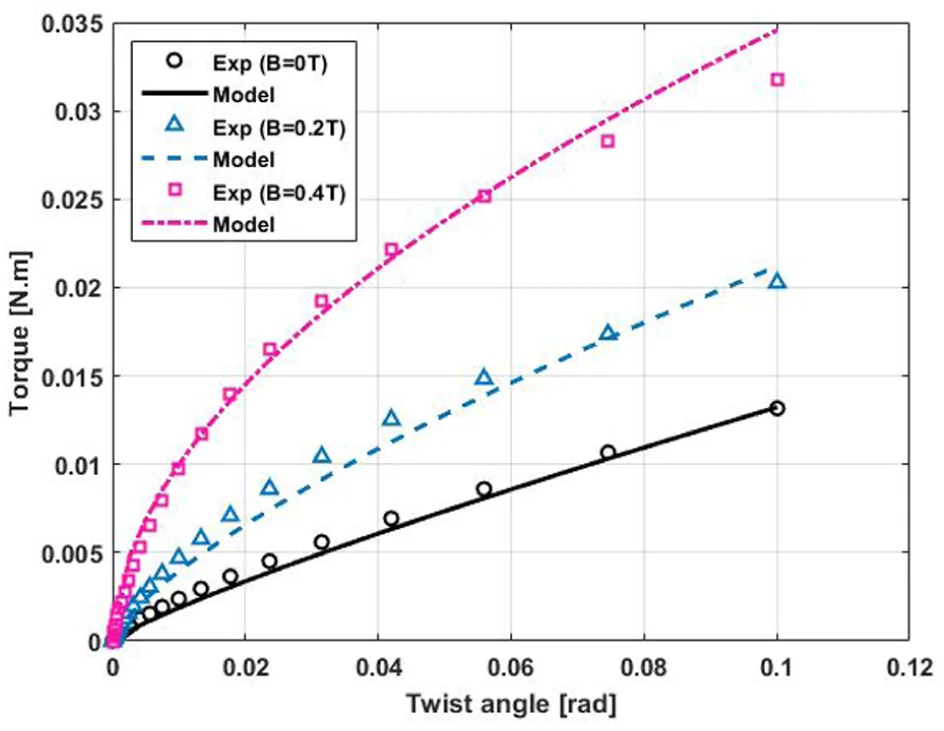
Comparison of torque-twist behavior obtained from proposed model with experimental data at $T_{b}=21{}^{\circ}C$ and axial particle chain orientation.

To obtain the total stress tensor, the constitutive relations introduced earlier are applied, yielding:

(20) $$\begin{array}{c} \sigma_{\mathrm{tot}}=\rho F{\left(\frac{\partial \psi }{\partial F}\right)}^{T}+\mu_{0}^{-1}\left[B⊗B-\frac{1}{2}\;\left(B.B\right)I\right]+\left(M.B\right)I\\ \;-B⊗M-pI \end{array}$$

where term vacuum Maxwell stress is introduced in which $\mu_{0}$ is the magnetic permeability of free space and has the value of $4\pi \times 10^{-7}\frac{H}{m}\;$ , I is the isotropic unit rank-2 tensor, p is the Lagrange multiplier for incompressibility condition, and M is the magnetization vector. With all required relations established, the formulation can now be applied to a boundary value problem involving a transversely isotropic MRE cylinder whose particle-chain orientation is denoted by unit vector A . The specimen is subjected to combined mechanical, magnetic, and thermal loadings. Mechanically, the bottom face of the cylinder is fixed, while the top face is subjected to a prescribed twist. The magnetic induction is applied along the z -axis, coinciding with the particle-chain orientation. Thermal loading is imposed by prescribing a fixed temperature at the bottom surface of the cylinder. Given the mapping between the reference and current configurations, the deformation gradient and the corresponding right Cauchy–Green deformation tensor can be expressed as follows:

(21) $$\begin{array}{c} F=\lambda_{z}^{-1/2}\cos\;\left({\mathrm{kX}}_{3}\right)e_{1}e_{1}-\lambda_{z}^{-1/2}\sin\;\left({\mathrm{kX}}_{3}\right)e_{1}e_{2}\\ \;-\lambda_{z}^{-1/2}{\mathrm{kx}}_{2}e_{1}e_{3}+\lambda_{z}^{-1/2}\sin\;\left({\mathrm{kX}}_{3}\right)e_{2}e_{1}\\ \;+\lambda_{z}^{-1/2}\cos\;\left({\mathrm{kX}}_{3}\right)e_{2}e_{2}+\lambda_{z}^{-1/2}{\mathrm{kx}}_{1}e_{2}e_{3}+\lambda_{z}e_{3}e_{3} \end{array}$$

(22) $$\begin{array}{c} c=\lambda_{z}^{-1}e_{1}e_{1}-\lambda_{z}^{-1}{\mathrm{kX}}_{2}e_{1}e_{3}+\lambda_{z}^{-1}e_{2}e_{2}\\ \;+\lambda_{z}^{-1}{\mathrm{kX}}_{1}e_{2}e_{3}-\lambda_{z}^{-1}{\mathrm{kX}}_{2}e_{3}e_{1}\\ \;+\lambda_{z}^{-1}{\mathrm{kX}}_{1}e_{3}e_{2}+\lambda_{z}^{-1}(k^{2}X_{1}^{2}+k^{2}X_{2}^{2}+\lambda_{z}^{3})e_{3}e_{3} \end{array}$$

here, k denotes the twist angle per unit length. By substituting the total free energy function ψ together with equation (16), the stress field within the MRE sample can be expressed as a function of the independent variables. Applying the appropriate boundary conditions then allows the torque on the top surface of the cylinder to be determined. To validate the proposed model and to identify the corresponding material constants, the predicted torque–twist response is compared with the experimental results (Figure 6). For parameter identification, a nonlinear least-squares optimization procedure based on the Trust-Region-Reflective algorithm is employed. The identified values of the material constants are summarized in Table 3. Regarding the validation and generality of proposed model, it should be emphasized that the formulation is grounded in a continuum-mechanics framework combined with finite strain theory, which is capable of describing large deformations. Moreover, the constitutive behavior is derived from a Helmholtz free energy density and laws of thermodynamics that are defined independently of the specimen geometry. As a result, variations in sample dimensions, such as height or diameter, do not alter the constitutive response itself, but only affect the problem through changes in the boundary conditions and input parameters. Consequently, as long as the deformation gradient tensor F and other boundary conditions are defined correctly, the proposed modeling framework is applicable to specimens with arbitrary geometries, provided that the underlying modeling assumptions remain valid.

**Table 3.** The model parameter identification.

$C_{12}$ ( kPa )	$C_{22}$ ( Pa )	$\gamma_{1}$ ( Pa )	$\gamma_{2}$ ( Pa )
$209.6\times 10^{2}$	885.0×10	$983.04\times 10^{2}$	$198.13\times 10^{2}$

**Figure 7.**
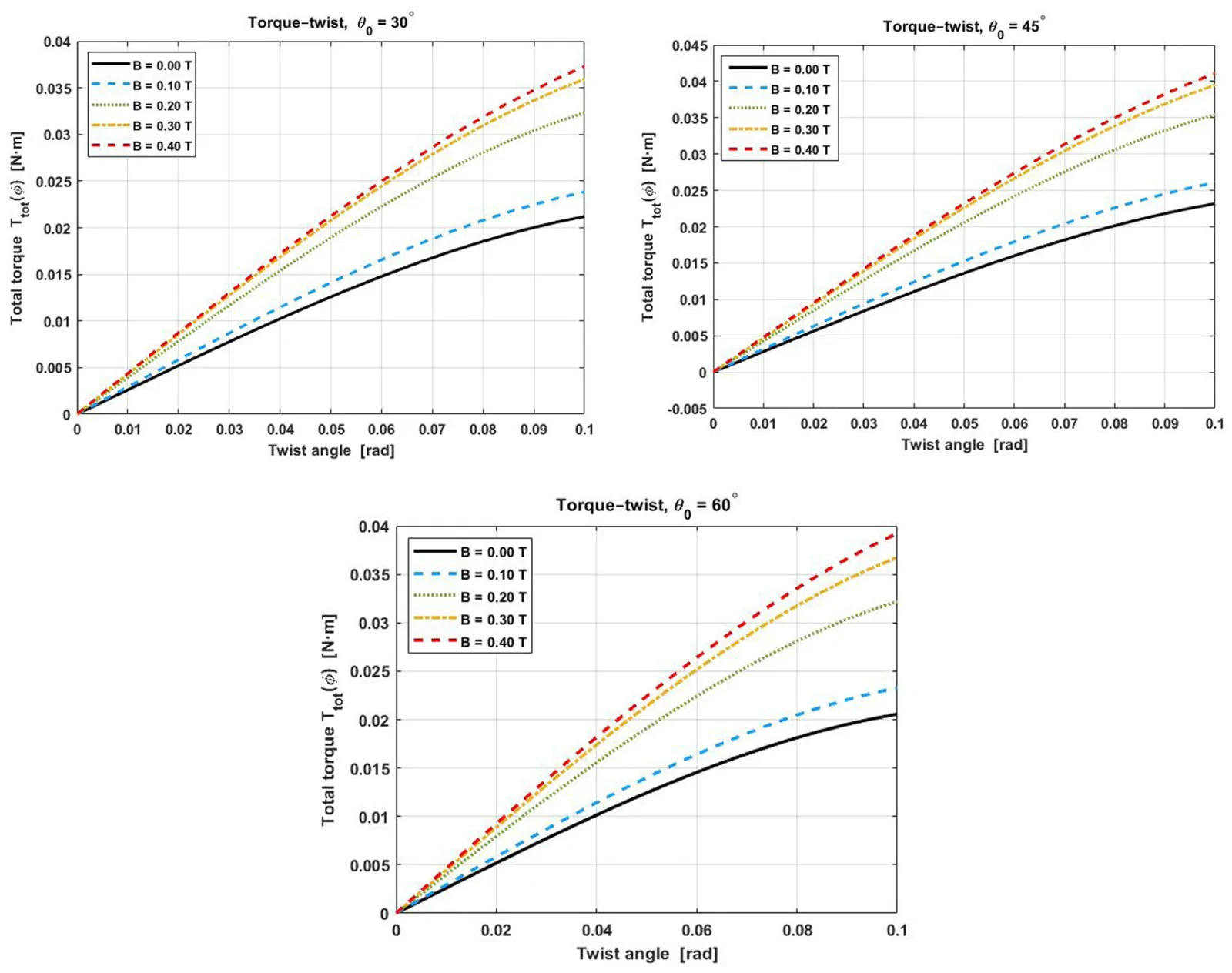
Torque-twist curves transversely isotropic MRE with different chain angle under different magnetic inductions at $T_{b}=30{}^{\circ}C$ .

With the model verified and the material parameters identified, additional analyses are carried out to investigate the torque–twist response of the transversely isotropic MRE specimens with different particle-chain orientation under different loading conditions. Regarding the different particle-chain orientation, the parameter $\theta_{0}$ is denoted as the mean dominant chain direction angle with respect to cylinder axis where the model does not assume multiple chain families. Results for total torque versus twist angle under varying magnetic flux densities considering a base temperature of $T_{b}=30{}^{\circ}C$ and for different particle-chain angles of 30°, 45°, and 60° relative to the axial direction, are then presented below in Figure 7.

**Figure 8.**
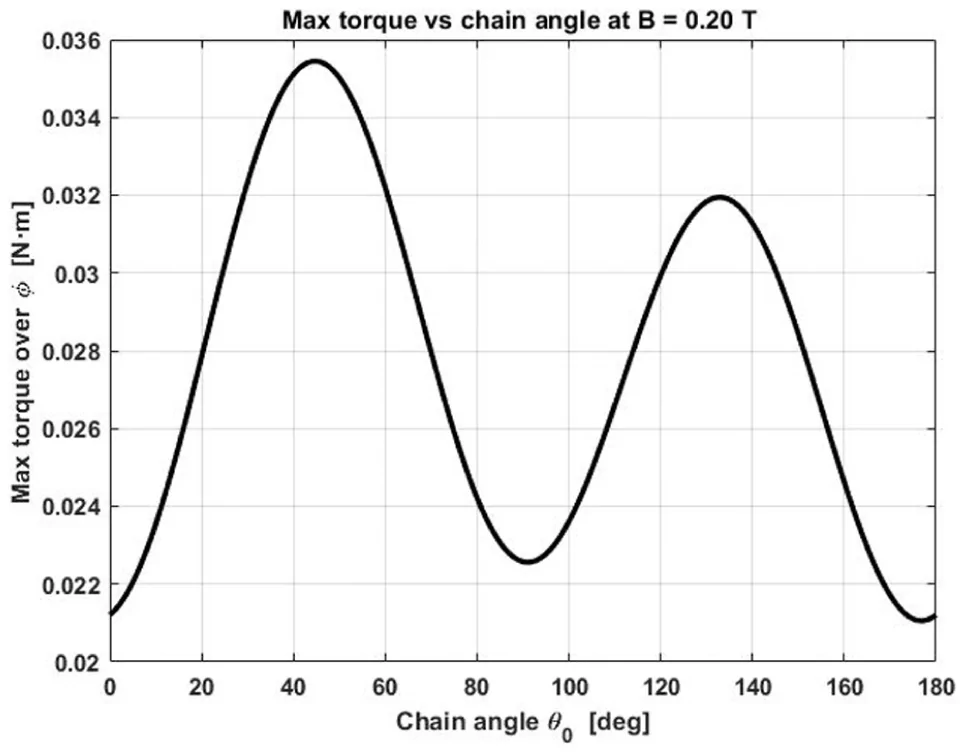
Max torque required at twist angle of 0.1 rad at fixed B=0.2T and $T_{b}=30{}^{\circ}C$ .

Results show that increasing the particle-chain angle with respect to the cylinder axis leads to a higher stiffness up to approximately 45°. Beyond this angle, the trend reverses, and the stiffness decreases with further increases in the chain orientation. To provide a clearer understanding of the influence of chain angle on the overall torque–twist response of the transversely isotropic MRE, the maximum torque required to twist the top surface of the cylinder to a fixed twist level is plotted over a wider range of chain orientations, as shown in Figure 8. According to this figure, in torsional loading of a transversely isotropic MRE with negligible magnetostriction and angle-independent magnetic stiffening, the maximum torque $T_{\max}$ varies with the chain orientation $\theta_{0}$ and exhibits two distinct peaks near 45° and 135°. This behavior arises from the fact that torsion resolves into principal tensile and compressive directions that are oriented at 45° relative to the shear directions. Specifically, at each material point, torsional deformation induces a state of simple shear in the $\left(e_{\theta },e_{z}\right)$ plane, in which the locally tensile and compressive directions do not coincide with the shear axes but instead correspond to the principal strain (or principal stretch) directions within the same $\left(e_{\theta },e_{z}\right)$ plane. As a result, the fiber-related invariants appearing in the energy function satisfy $I_{7}-1=\gamma \cdot \sin(2\theta_{0})+(\gamma^{2}/2)[1\;-\cos(2\theta_{0})]\;$ and $2I_{8}\;-I_{7}\;-1\;=3\gamma \cdot \sin(2\theta_{0})\;+(3\gamma^{2}/2)[1\;-\cos(2\theta_{0})]$ , when these invariants are squared within the energy function, they produce a dominant quadratic term proportional to $\left(\gamma_{1}\;+\;9\gamma_{2})\gamma^{2}\cdot \sin^{2}(2\theta_{0}\right)$ which attains its maximum when $\sin\left(2\theta_{0}\right)=1$ , corresponding to orientations near ±45°. At finite twist amplitudes, the same invariants generate additional cubic contributions proportional to $\gamma^{3}\cdot \sin(2\theta_{0})[1\;-\cos(2\theta_{0})]$ which are positive for 0° < θ < 90° and negative for $90^{{}^{\circ}}<\theta_{0}<180^{{}^{\circ}}$ . This sign change explains the presence of the two maxima near 45° and 135° in the torque–orientation response with the first peak being more pronounced.

## Conclusion

In this study, a physics-based constitutive model based on nonlinear continuum mechanics was developed to predict the quasi-static magneto–mechanical–thermal behavior of transversely isotropic MREs. The effect of temperature was incorporated into the proposed model in two complementary ways. First, a temperature-dependent initial structural stiffness was introduced to account for the steady-state influence of temperature on the material response. Second, by employing the assumption of constant specific heat capacity, temperature was explicitly included in the total Helmholtz free energy function. This formulation enables the model to capture the influence of temperature gradients within the MRE sample. The model was then validated using experimental torque–twist data obtained from a cylindrical transversely isotropic MRE specimen subjected to magnetic induction levels ranging from 0 to 0.4 T and temperatures between −10°C and 40°C. Following validation, the material parameters were identified, and the model was used to examine the response under various particle-chain orientations. The results show that, for the fabricated MRE considered here, the stiffness increases with both magnetic induction and temperature. In addition, the torque–twist response exhibits two distinct maxima at chain angles of approximately 45° and 135° relative to the cylinder axis. These findings demonstrate that the proposed model is capable of accurately capturing the coupled behavior of transversely isotropic MREs under combined mechanical, magnetic, and thermal loading conditions, making it a useful tool in the early stages of magnetoactive device and structure design.
